# Prenatal Developmental Trajectories of Fluctuating Asymmetry in Bat Humeri

**DOI:** 10.3389/fcell.2021.639522

**Published:** 2021-05-26

**Authors:** Camilo López-Aguirre, Suzanne J. Hand, Daisuke Koyabu, Vuong Tan Tu, Laura A. B. Wilson

**Affiliations:** ^1^Department of Anthropology, University of Toronto Scarborough, Toronto, ON, Canada; ^2^Earth and Sustainability Science Research Centre, School of Biological, Earth and Environmental Sciences, University of New South Wales, Sydney, NSW, Australia; ^3^Jockey Club College of Veterinary Medicine and Life Sciences, City University of Hong Kong, Kowloon, Hong Kong; ^4^Research and Development Center for Precision Medicine, University of Tsukuba, Tsukuba, Japan; ^5^Institute of Ecology and Biological Resources, Vietnam Academy of Science and Technology, Hanoi, Vietnam; ^6^Graduate University of Science and Technology, Vietnam Academy of Science and Technology, Hanoi, Vietnam; ^7^School of Archaeology and Anthropology, The Australian National University, Canberra, ACT, Australia

**Keywords:** fluctuating asymmetry, Chiroptera, prenatal development, humerus, morphogenesis

## Abstract

Fluctuating asymmetry (random fluctuations between the left and right sides of the body) has been interpreted as an index to quantify both the developmental instabilities and homeostatic capabilities of organisms, linking the phenotypic and genotypic aspects of morphogenesis. However, studying the ontogenesis of fluctuating asymmetry has been limited to mostly model organisms in postnatal stages, missing prenatal trajectories of asymmetry that could better elucidate decoupled developmental pathways controlling symmetric bone elongation and thickening. In this study, we quantified the presence and magnitude of asymmetry during the prenatal development of bats, focusing on the humerus, a highly specialized bone adapted in bats to perform under multiple functional demands. We deconstructed levels of asymmetry by measuring the longitudinal and cross-sectional asymmetry of the humerus using a combination of linear measurements and geometric morphometrics. We tested the presence of different types of asymmetry and calculated the magnitude of size-controlled fluctuating asymmetry to assess developmental instability. Statistical support for the presence of fluctuating asymmetry was found for both longitudinal and cross-sectional asymmetry, explaining on average 16% of asymmetric variation. Significant directional asymmetry accounted for less than 6.6% of asymmetric variation. Both measures of fluctuating asymmetry remained relatively stable throughout ontogeny, but cross-sectional asymmetry was significantly different across developmental stages. Finally, we did not find a correspondence between developmental patterns of longitudinal and cross-sectional asymmetry, indicating that processes promoting symmetrical bone elongation and thickening work independently. We suggest various functional pressures linked to newborn bats’ ecology associated with longitudinal (altricial flight capabilities) and cross-sectional (precocial clinging ability) developmental asymmetry differentially. We hypothesize that stable magnitudes of fluctuating asymmetry across development could indicate the presence of developmental mechanisms buffering developmental instability.

## Introduction

Bats (Chiroptera) have a highly specialized postcranial morphology adapted to perform self-powered flight, a trait unique among mammals. Compared to terrestrial mammals, the forelimb skeleton of bats is characterized by aerial-based functional adaptations that provide structural support to the wing (bone elongation of distal bones) (Panyutina et al., 2015), withstand biomechanical loading (increased bone density) (Swartz, 1997; Dumont, 2010), enhance flight maneuverability (proximo-distal demineralization) (Papadimitriou et al., 1996), and facilitate the generation of flight power (increase of muscle mass in the shoulder girdle) (Panyutina et al., 2015). Moreover, morphological adaptations related to different functional pressures can occur within a single bone (e.g., increased muscle attachment area and biomechanical load resistance in the humerus; Panyutina et al., 2015). Such a variety of long bone adaptations arise ontogenically, mainly through two bone remodeling processes: bone elongation and bone thickening (Enlow, 1963). The former is linked to axial bone deposition *via* endochondral ossification at growth plates (Webber et al., 2015), whereas the latter results from endosteal and periosteal bone deposition *via* intramembranous ossification in the periosteum/endosteum (Kronenberg, 2003). These two processes derive from independent morphogenetic pathways that respond to different factors, indicating decoupled developmental trajectories controlling the optimal development of individual forelimb bones (Atchley and Hall, 1991; Montoya-Sanhueza et al., 2019). However, our understanding of the differences in developmental trajectories of bone thickening and elongation in the bat wing remains largely limited.

Uncovering the historical trajectories that led to the morphological diversification and specialization of bats has been greatly limited by a markedly incomplete fossil record (Brown et al., 2019). Evolutionary developmental biology has emerged as a promising approach to study the evolution of bats while circumventing limitations in the fossil record (Adams, 2008; Cooper et al., 2012; Camacho et al., 2019; López-Aguirre et al., 2019,a,b). Studies have provided evidence for the ontogenetic mechanisms behind forelimb specialization in bats and the evolution of vertebrate flight (Sears et al., 2006; Adams, 2008; Cretekos et al., 2008; Farnum et al., 2008; Cooper et al., 2012; Adams and Shaw, 2013), ecology-driven deviations in chiropteran development from general mammalian patterns, and the phylogenetic signal in postcranial development (Adams, 1992; Koyabu and Son, 2014; López-Aguirre et al., 2019,a,b).

For body plans that are naturally symmetrical, deviations from an “ideal” state have been interpreted as a signal of reduced fitness (Dongen, 2006). Accordingly, quantifying the deviation from perfect symmetry can indicate the amount of stress an organism undergoes and its homeostatic capacity (i.e., buffering of instabilities to maintain fitness) (Gummer and Brigham, 1995; Aparicio and Bonal, 2002). The main regulatory mechanisms that influence phenotypic symmetry occur ontogenically, when the genotypic and phenotypic mechanisms involved in morphogenesis can be destabilized by genetic or environmental stressors (i.e., developmental noise) (Hallgrímsson, 1998, 1999; Hallgrímsson et al., 2003; Kellner and Alford, 2003). Evolutionary studies have provided evidence for the heritability of an organism’s capacity to buffer developmental noise (developmental stability/instability, DI), suggesting that natural selection can act as a regulator of phenotypic asymmetry (Tocts et al., 2016).

Phenotypic asymmetry in animals with bilateral asymmetry can be quantified by computing the morphometric differences between the right and the left side of the body (right–left, R–L) (Palmer, 1994). Advances in theoretical framework to quantify body asymmetry has enabled the recognition of three types of asymmetry, each with a different biological interpretation: fluctuating asymmetry (FA) is characterized as random deviations from “ideal” perfectly symmetrical phenotypes, directional asymmetry (DA) is described as a natural tendency to have consistently asymmetrical phenotypes (i.e., one side always larger than the other), and antisymmetry (AS) represents a pattern where symmetrical phenotypes are least favored and asymmetry is equally distributed across both sides (Klingenberg, 2015). All three types of asymmetry have also been described in mathematical terms: FA is characterized by a normal distribution of asymmetry values (R–L) along a value mean of zero, DA is described by a normal distribution of asymmetry values along a mean different to zero, and AS is identified where asymmetry values have a bimodal distribution and most values are different from zero (Palmer, 1994; Klingenberg, 2015). Combining the biological and mathematical interpretations of phenotypic asymmetry, FA has been regularly used as a possible indicator of DI, although some studies argue that DA and AS can also be indicators of DI (Palmer, 1994; Leamy and Klingenberg, 2005). Despite the utility of studying FA and DI, the efficacy of the theoretical framework traditionally applied to detect a real FA–DI link has been debated (Palmer, 1994; Dongen, 2006; Klingenberg, 2015).

The FA–DI link can vary in response to ecological, genetic, environmental, and developmental factors, stressing the need to study it at multiple scales to test a variety of hypotheses (Kellner and Alford, 2003). Swaddle and Witter (1997) summarized and Kellner and Alford (2003) postulated and tested predictions for a list of hypotheses on the ontogeny of FA describing developmental patterns of asymmetry and possible evolutionary mechanisms shaping them. These include the following: small fluctuations during early growth are magnified during later morphogenesis (i.e., magnification of asymmetry hypothesis), side-biased environmental influences can skew growth toward asymmetrical phenotypes (i.e., directional external cues hypothesis), accumulative growth of independent subunits will homogenize morphogenesis, reducing asymmetry throughout development (i.e., coin toss hypothesis), and developmental feedback mechanisms will stabilize asymmetric growth between structures by either promoting or constraining growth (i.e., compensatory growth hypothesis). Studies on the developmental basis of FA have also been restricted in scope (mostly focused on postnatal development) and study groups (invertebrates and captive populations) (Hallgrímsson, 1999; Hallgrímsson et al., 2003; Leamy and Klingenberg, 2005; Blackburn, 2011; Perchalski et al., 2018), limiting our understanding of variation in wild non-model species and potential insights into the mechanisms controlling FA in early development. Based on the limited studies available, decreasing magnitudes of FA across prenatal development have been reported as a result of the interaction between variations in timing of growth and growth rates (Hallgrímsson, 1998; Hallgrímsson et al., 2003; Kellner and Alford, 2003). In contrast, magnitudes of FA have been reported to increase during the development of the mammalian skeleton due to cumulative variability in growth regulation and/or bone remodeling (i.e., morphogenetic drift model; Hallgrímsson, 1998).

Asymmetry in bats has been studied at the cranial and postcranial level (Juste et al., 2001a,b; Lüpold et al., 2004; Voigt et al., 2005; López-Aguirre and Pérez-Torres, 2015), all studies being based on the analysis of adult specimens only. Forelimb FA has been associated with differential reproductive success (Lüpold et al., 2004; Voigt et al., 2005), suggesting that sexual selection favors symmetric individuals in *Saccopteryx bilineata* (Voigt et al., 2005) and a significant link between asymmetry and reproductive potential in *Carollia perspicillata* (Monteiro et al., 2019). A correlation between wing FA and resistance to environmental stress and resilience to anthropogenic habitat change has also been assessed, suggesting high resilience in Neotropical bat species (de Figueiredo et al., 2015; Castillo-Figueroa, 2018). Compensatory growth has been reported in the wing of the vampire bat *Desmodus rotundus* as a way to maintain wingspan symmetry (Ueti et al., 2015), while sex-based differences in the magnitudes of FA have been reported in the wing of *D. rotundus* and the cranium of *Artibeus lituratus* (López-Aguirre and Pérez-Torres, 2015; Ueti et al., 2015). Research also indicates that variation in the levels of FA across morphological (both cranial and postcranial) traits could depend on functional importance, with FA decreasing in traits under higher functional demands for feeding and locomotion (Gummer and Brigham, 1995; López-Aguirre and Pérez-Torres, 2015; Robaina et al., 2017). Despite the repeated study of bat forelimb FA, its ontogenetic basis and how it varies across different bone growth dimensions (i.e., bone elongation and thickening) remain unknown.

The objective of this study was to assess whether the ontogenetic trajectories of phenotypic asymmetry during bone elongation and thickening in bats are decoupled. We analyzed the presence and magnitude of FA in the prenatal morphogenesis of the humerus in bats, representing the first developmental study of FA in Chiroptera. We focused on the humerus because it represents a clear example of multiple functional demands acting on a single bone (i.e., withstanding torsional and bending stress, increasing muscle insertion area, and controlling the maneuverability of the wing) (Swartz et al., 1992; Panyutina et al., 2015). Humeral cross-sectional shape has been found to reflect foraging differences across bat taxa (López-Aguirre et al., 2019). Furthermore, prenatal limb FA has been described as an accurate indicator of DI in human fetuses (Klingenberg and Nijhout, 1999; Broek et al., 2017). We quantify asymmetry based on bone elongation and cross-sectional cortical bone deposition as a way to exemplify the multipatterned process of bone growth (Klingenberg and McIntyre, 1998). Humeral length asymmetry is commonly used in bat studies (Gummer and Brigham, 1995; Voigt et al., 2005; de Figueiredo et al., 2015; Ueti et al., 2015; Robaina et al., 2017; Castillo-Figueroa, 2018), whereas cross-sectional asymmetry is commonly measured in other mammals (Macintosh et al., 2013; Wilson and Humphrey, 2015; Perchalski et al., 2018). Based on previous studies inferring compensatory growth in the wing of bats and previous evidence of decreasing FA across development in terrestrial mammals (Hallgrímsson et al., 2003; Ueti et al., 2015), we hypothesize that asymmetry of the humerus will decrease throughout ontogeny. Because bone thickening and elongation in mammals represent independent morphogenetic trajectories, we predict that cross-sectional and longitudinal asymmetry will not be correlated.

## Materials and Methods

### Sampling

**FIGURE 1:**
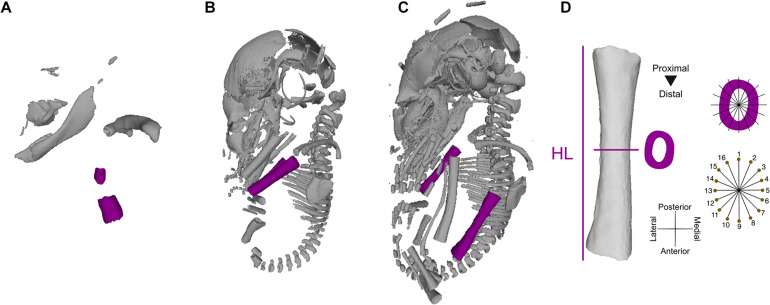
3D virtual models of ontogenetic series of *Rhinolophus thomasii*, representing the samples from which raw measurements were taken. Stage 1 **(A)**, stage 6 **(B)**, and stage 9 **(C)**. Landmarking and measuring protocol used to quantify humeral length (HL) and cortical thickness **(D)**.

A total of 66 prenatal specimens from 11 bat species (Table 1, see Figures 1A–C) were collected through taxonomic fieldwork in Vietnam by VTT and DK under collection permit no. 972/UBND-TH issued by Tuyen Quang Provincial People’s Committee and research and ethics permit no. 322/STTNSV of the Institute of Ecology and Biological Resources, Vietnam Academy of Sciences. All specimens were fixed in Serra’s fixative (ethanol, formalin, and glacial acetic acid mixed 6:3:1 by volume) for 48 h, then transferred, and preserved in 70% ethanol. 3D scanning of the embryos and fetuses was performed using a microfocal X−ray computed tomography system at the University Museum, University of Tokyo (TXS225−ACTIS; TESCO; Tokyo, Japan), with 70-kV source voltage and 114 μA source currents at a resolution of 36 μm. All osseous skeletal elements were segmented by the first author using the thresholding tool and the predetermined bone setting in MIMICS v. 20 software (Materialise NV, Leuven, Belgium). To standardize finer manual segmentation in early embryos, thresholding of Hounsfield unit values of osseous tissue was performed using the half-maximum height method (i.e., gradual change in computerized tomography values at the boundary of a structure) (Spoor et al., 1993). This method homogenously retrieved diaphyseal osseous tissue only, removing segmenting inconsistencies related to the imaging of diaphyseal cartilaginous tissue. Smoothing techniques were not used to prevent artificially changing the dimensions of the periosteal surface of the models. After selecting specimens with at least partially ossified and unbroken humeri, three individuals were excluded from the final sample. The left and right humeri were segmented and exported as STL files for further processing and analysis.

**TABLE 1:** Composition of the sample used in this study, including the number of specimens and the developmental stages per species.

Family	Species	*N*	Developmental stages
Hipposideridae	*Aselliscus dongbacana*	14	4, 6–10
Hipposideridae	*Aselliscus stoliczkanus*	13	4–5, 7–10
Pteropodidae	*Cynopterus sphinx*	4	2, 8, 10
Vespertilionidae	*Hesperoptenus blandfordi*	5	1, 4–5, 8
Hipposideridae	*Hipposideros larvatus*	4	7–9
Vespertilionidae	*Kerivoula hardwickii*	7	2–5
Miniopteridae	*Miniopterus schreibersii*	2	10
Vespertilionidae	*Myotis* sp.	2	4–5
Rhinolophidae	*Rhinolophus pearsonii*	1	4
Rhinolophidae	*Rhinolophus pusillus*	1	2
Rhinolophidae	*Rhinolophus thomasi*	10	1, 6–9

### Data Collection

Humeral development was described based on bone elongation (i.e., humeral length, HL) and cortical bone deposition (i.e., periosteal diameter as a proxy for cross-sectional cortical thickness, CT) (Figure 1D). To estimate cross-sectional and HL measurements, 3D humeri models were imported into Rhinoceros 5.0 (Robert McNeel & Associates, Seattle, WA). To remove the non-shape effects of translation, rotation, and scale, all humeri models were aligned to a standard position in 3D space, following a protocol for long-bone cross-sectional geometry (see Wilson and Humphrey, 2015). HL was automatically measured as the length of the long axis of a rectilinear box (i.e., bounding box) enclosing the model created using the *BoundingBox* command in Rhinoceros 5.0, preventing human measurement error (ME). Cross-sections of the left and right humeri were extracted at the midshaft (i.e., 50% of HL) for a total of 126 cross-sections [63 × 2 (left and right sides)]. The midshaft was extracted by placing a perpendicular axis intersecting the 3D model at the midpoint of the length of the rectilinear bounding box, dividing HL in half. Small deviations in the placing of the cross-sections could occur due to asymmetrical development of cartilaginous epiphyseal tissue. Humeral CT was quantified using a geometric morphometrics-based approach. Following the method described in Wilson and Humphrey (2015), we used a set of 16 equiangular landmarks semi-automatically placed along the periosteal surface of each cross-section (Figure 1D). The cross-sections were aligned with a set of 16 equiangular radii along the centroid of each cross-section, aligning the radii with the anatomical axes of the bone (e.g., radii 1 and 9 represent the antero-posterior axis). The landmarks were automatically placed on the intersection of individual radii and the periosteal surface of each cross-section. CT was quantified as the average of interlandmark distances between pairs of landmarks that formed linear axes (e.g., landmarks 1 and 9; see Figure 1D) using the interlmkdist function in R package Geomorph 3.2 (Adams et al., 2013). Our automated cross-sectional geometric morphometrics-based approach enables the quantification of CT while circumventing the lack of identifiable homologous landmarks early in prenatal development. To control for the effect of matching bilateral symmetry (i.e., the left and right sides of the body are mirror images), the landmark coordinates of the cross-sections of the right side were reflected along the antero-posterior axis by multiplying the coordinates of that axis by −1. Given the lack of Carnegie staging systems for many non-model taxa (nine of the 11 species in our sample), staging of developmental series was based on crown-to-rump length and bone ossification sequence as described in López-Aguirre et al. (2019a) and following the general patterns described in bat development (Cretekos et al., 2005). All developmental stages were represented by at least three individuals (Supplementary Table 1).

### Estimation of Asymmetry

Asymmetry in bilateral organisms can be described as the difference between both sides of the body (e.g., right–left) (Palmer, 1994), which we estimated based on HL (longitudinal asymmetry) and CT (cross-sectional asymmetry). Individual longitudinal and cross-sectional asymmetries were quantified as the signed difference between right and left HL, negative and positive values indicating the directionality of asymmetry (Palmer, 1994). Individual longitudinal FA was described as the size-corrected signed difference between sides, and per stage FA was calculated as the variance of individual FA measurements across a population (i.e., FA6 index) (Palmer, 1994). FA6 is a single-trait index that expresses FA as the variance in asymmetry proportional to trait size (i.e., HL) in an individual so as to truly represent DI and not developmental bone growth,

$$\text{var}\left[\frac{R-L}{0.5*\left(RL\right)}\right].$$

### Data Analysis

The presence of FA and DA in longitudinal and cross-sectional asymmetry was statistically tested with full-factorial ANOVAs using side (left or right), individual, and duplicate as factors (FA ∼ side + individual + side/individual; see Table 2; Monteiro et al., 2019; Rivera and Neely, 2020). The side factor provides a statistical test for DA, whereas the side–individual interaction provides statistical tests for FA. Measurement error was not computed because the automated protocol implemented to obtain HL and CT ensures that no human error could affect the measuring process.

**TABLE 2:** ANOVA statistical tests of significance of fluctuating asymmetry (FA) and directional asymmetry (DA) in cortical thickness (CT) and humeral length (HL).

	Df	SS	MS	Rsq	*F*	Pr (> *F*)
HL						
Individual	62	1063.9	17.160	0.9925	2304	<0.0001
Side	1	0	0	0	0	0.983
Individual*Side	62	0.8	0.013	0.0075	1.759	<0.05
Residuals	125	0.9	0.007	4.8E-30		
CT						
Individual	62	2.472E-05	3.987E-07	0.5511	6.484E+23	<0.0001
Side	1	9.500E-08	9.550E-08	0.1320	1.553E+23	<0.0001
Individual*Side	62	1.422E-05	2.293E-07	0.3169	3.730E+23	<0.0001
Residuals	125	0	0	0		

Side factor tests for DA, individual*side interaction tests for FA, and replicate tests for ME. Df, degrees of freedom; SS, sum of squares; MS, mean square; Rsq, R square.

Developmental trajectories of individual longitudinal and cross-sectional FA were explored using box plots, while per stage FA6 was explored using bar plots. Statistical differences across developmental stages were tested using two ANOVAs, using the stage of each individual as a factor and unsigned longitudinal and cross-sectional FA (longitudinal FA∼stage and cross-sectional FA∼stage).

We tested the association of longitudinal and cross-sectional FA across development using a linear regression model based on ordinary least squares. We used linear regression models to test the effect of the number of specimens and species in values of average longitudinal and cross-sectional FA per developmental stage to assess whether uneven sample composition affected our results. Using a subsample of the best sampled genus, we explored the association between peaks in humeral growth and peaks in magnitudes of asymmetry. Finally, we tested the consistency of our results by recreating the ontogenetic patterns of longitudinal and cross-sectional FA in a subsample of the best sampled species (*Aselliscus dongbacana* and *Aselliscus stoliczkanus*).

## Results

### Individual Longitudinal and Cross-Sectional Asymmetry

**FIGURE 2:**
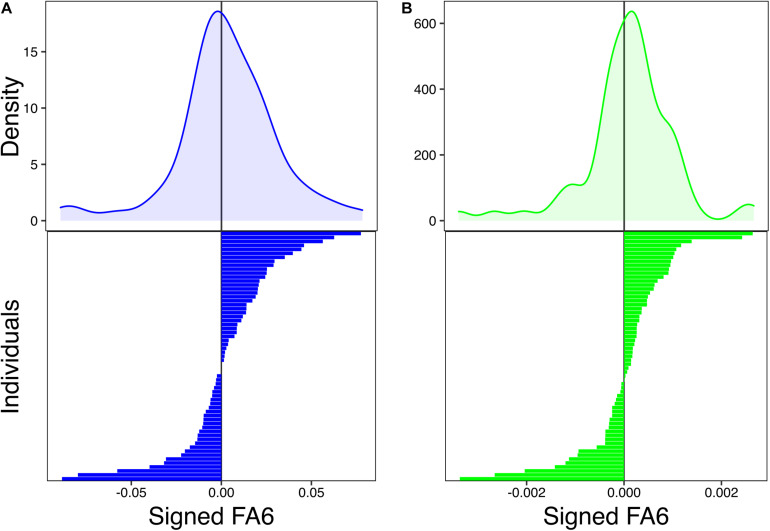
Probability density functions of distribution of values of longitudinal (signed fluctuating asymmetry, FA) **(A)** and cross-sectional FA (signed FA) **(B)**. Bar plots show the individual asymmetry values from which density functions were estimated.

Distribution of individuals’ values of longitudinal and cross-sectional FA demonstrate a normal distribution with a mean near zero (Figure 2), supporting the presence of FA in longitudinal and cross-sectional humeral asymmetry throughout development. Moreover, 42.86% of individuals had negative values of longitudinal FA (Figure 2A), indicating that a narrow majority of individuals had larger right humeri (57.14%). Furthermore, 39.68% of individuals showed negative values of cross-sectional asymmetry (Figure 2B). Across datasets (cross-sectional and HL), only three individuals were found to have perfectly symmetrical humeri (i.e., R–L = 0) for HL in developmental stages 5, 7, and 8.

**FIGURE 3:**
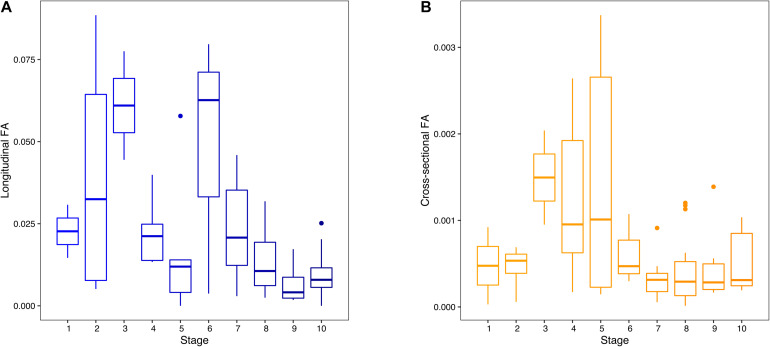
Box plots of individual longitudinal (unsigned fluctuating asymmetry, FA) **(A)** and cross-sectional (unsigned FA) **(B)** FA values across development. Stages 1–10 represent early to late prenatal development.

Box plots of individuals’ longitudinal and cross-sectional FA values illustrated independent trajectories of cross-sectional and longitudinal humeral FA across development (Figure 3). Unsigned longitudinal FA indicated two peaks of high values of longitudinal FA in individuals early in prenatal development (stages 2, 3, and 6), separated by a sharp decrease in longitudinal FA values between stages 4 and 5. Longitudinal FA values steadily decreased from stage 7 onwards, with a noticeable increase in dispersion in stages 2 and 6 (Figure 3A). A similar pattern was found in our subsample of the best sampled species, with relatively stable longitudinal FA after stage 1 and an increase in variability in stages 8 and 9 (Supplementary Figure 1). Cross-sectional FA showed low magnitudes of FA in individuals in the early stages of prenatal development (stages 1 and 2), followed by an increase between stages 3 and 5 (Figure 3B). Individuals in stage 5 showed the highest dispersion of FA values. Cross-sectional FA consistently decreased from stage 6 onwards. The cross-sectional FA in our subsample remained relatively stable after stage 3, followed by a decrease from stage 7, similar to our general results (Supplementary Figure 1).

### Presence and Magnitude of Fluctuating Asymmetry

The ANOVA of asymmetry on HL supported the presence of both DA and FA in longitudinal asymmetry (Table 2). Nevertheless, FA accounted for only 0.007% of variation, DA for less than 0.0001% of the variation, and individual variation for 99.25% of variability in raw HL. The markedly low values of longitudinal FA suggest that the extraction of the cross-sections did not vary due to asymmetrical growth rates between epiphyseal plates. The ANOVA of cross-sectional asymmetry found statistical support for the presence of FA and DA (Table 2). DA accounted for only 13.20% of cross-sectional variation and FA for 31.69% of variation, respectively. Individual variation explained the highest proportion of variation, accounting for 55.11% of cross-sectional asymmetrical variation.

### Developmental Trajectories of Fluctuating Asymmetry

**FIGURE 4:**
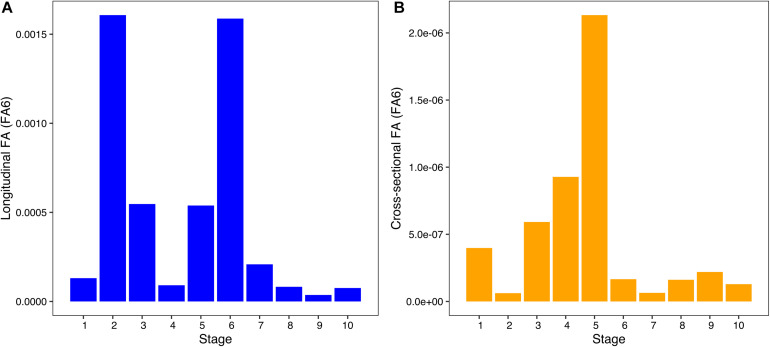
Bar plot of per stage longitudinal **(A)** and cross-sectional **(B)** FA6 values across development. Stages 1–10 represent early to late prenatal development.

The per-stage longitudinal and cross-sectional FA (FA6) indicated disparate trajectories across development (Figure 4). The longitudinal FA values showed two peaks (Figure 4A): one early in development (stage 2) and another in intermediate prenatal development (stage 6). The lowest longitudinal FA values were found in stages 4 and 8–10. Cross-sectional FA showed a single peak during mid-prenatal development (stage 5), followed by a significant decrease from stages 6 to 10 (Figure 4B). Despite the differences in the trajectories of longitudinal and cross-sectional FA throughout development, our results indicate a trend of stable FA throughout development. The ANOVAs for differences in longitudinal and cross-sectional FA across developmental stages found statistically significant differences in cross-sectional FA but not in longitudinal FA (Table 3). Our results do not reveal clear similarities between peaks of FA and peaks of humeral growth, indicating that asymmetry does not increase when growth rates are higher (Supplementary Figure 2). Nevertheless, future studies should further explore the possible link between growth rates and magnitudes of asymmetry during development.

**TABLE 3:** ANOVA test results for statistically significant differences in longitudinal and cross-sectional humeral fluctuating asymmetry (FA) across developmental stages.

	Df	SS	MS	Rsq	*F*	Pr (>*F*)
Longitudinal FA	1	0	0	−0.0164	0	0.993
Cross-sectional FA	1	2.1303E-06	2.1303E-06	0.0701	4.598	0.035

**Df, Degrees of freedom; SS, Sum of squares; MS, Mean square; Rsq, R square.**

**FIGURE 5:**
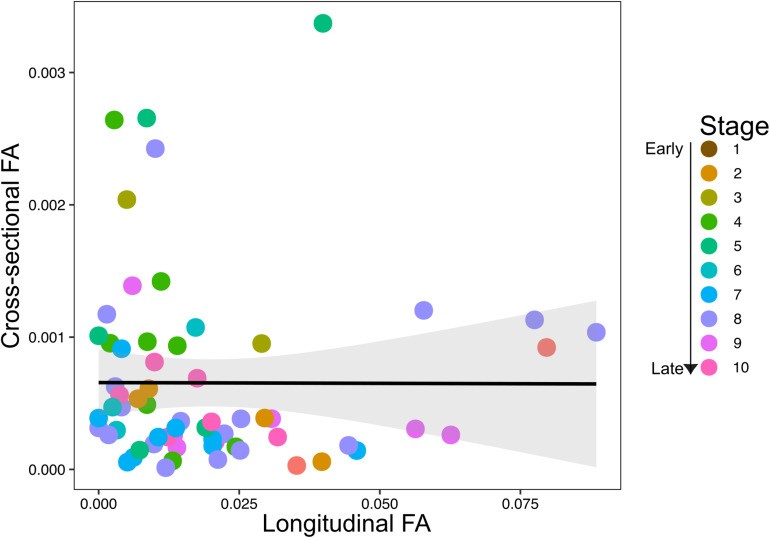
Scatterplot of association between longitudinal (unsigned fluctuating asymmetry, FA) and cross-sectional (unsigned FA) fluctuating asymmetry across prenatal development. Colored dots represent developmental stages, with stages 1–10 representing early to late prenatal development.

The scatterplots of longitudinal and cross-sectional asymmetry did not indicate a clear association between cross-sectional and longitudinal FA throughout development, with a slightly negative tendency (Figure 5). A linear regression model of cross-sectional and longitudinal FA confirmed a non-significant negative correlation between both dimensions of humeral FA (*R*^2^ = 0.016, *P* = 0.979). The cross-sectional and longitudinal FA also did not correlate with the number of specimens and species per stage (cross-sectional FA: specimens *R*^2^ = 0.136, *P* = 0.159 and species *R*^2^ = 0.183, *P* = 0.121; longitudinal FA: specimens *R*^2^ = 0.096, *P* = 0.66 and species *R*^2^ = 0.072, *P* = 0.546), indicating that sampling heterogeneity did not influence our results.

## Discussion

This study is the first to demonstrate the presence of FA and DA during bat prenatal development. Our results showed stable magnitudes of longitudinal and cross-sectional FA across prenatal development, rejecting our hypothesis of decreasing asymmetry throughout ontogeny. However, our results also show that both measures of FA (longitudinal and cross-sectional) did not correlate across development, showing decoupled ontogenetic trajectories, similar to our prediction. Decoupled longitudinal and cross-sectional asymmetry magnitudes also indicate that the developmental pathways regulating symmetrical bone elongation and thickening likely act independently within a single bone. Signaling pathways regulating symmetric growth have been detected in the apical ectodermal ridge and the zone of polarizing activity of the limb bud (Allard and Tabin, 2009; Wolpert, 2010). Sonic hedgehog and fibroblast growth signaling factors play a major role during limb growth, controlling limb polarity (i.e., anterior–posterior limb differentiation), cell proliferation, and symmetric growth (Allard and Tabin, 2009; Wolpert, 2010). Previous studies have found decreasing trajectories of asymmetry across prenatal development in the postcranium of humans and mice, suggesting an interplay between the timing of morphogenesis and growth rates over the course of ontogeny (Hallgrímsson, 1998; Hallgrímsson et al., 2003). The compensatory growth between left and right sides of the body in response to increased asymmetry has been discussed as a mechanism to reduce DI (Aparicio, 1998; Ueti et al., 2015).

FA in bats has been an area of increasing interest in recent decades (Gummer and Brigham, 1995; Juste et al., 2001a,b; Lüpold et al., 2004; Voigt et al., 2005; de Figueiredo et al., 2015; López-Aguirre and Pérez-Torres, 2015; Ueti et al., 2015; Robaina et al., 2017; Castillo-Figueroa, 2018; Monteiro et al., 2019). A set of two studies explored the patterns of FA in insular populations of fruit bat species *Eidolon helvum* and *Rousettus egyptiacus* of the Gulf of Guinea (Juste et al., 2001a,b). Juste et al. (2001a) found similar patterns of FA across populations of both species, discussing the interpretation of population asymmetry parameters (i.e., consistent patterns of FA for a set of characters across populations of the same species), and suggested its scalability above the species level. Juste et al. (2001b) found consistent magnitudes of multivariate FA and a significant integration of asymmetry across traits and species, hypothesizing high canalization in the developmental pathways controlling phenotypic asymmetry that are shared between the two species.

The presence and magnitude of FA has also been associated with reproductive success and sexual selection in bats (Voigt et al., 2005; Monteiro et al., 2019). Analyzing forearm length asymmetry, it has been suggested that sexual selection favors more symmetrical males in the polygynous greater sac-winged bat *Saccopteryx bilineata*, canalizing FA (Voigt et al., 2005). The number of offspring produced by males of *S. bilineata* was found to significantly decrease with increasing forearm asymmetry (Voigt et al., 2005). Increases in forearm asymmetry in the Neotropical frugivorous bat *Carollia perspicillata* have also been linked with a significant decrease in survival probability and the probability of more than one pregnancy per reproductive season (Monteiro et al., 2019). However, Lüpold et al. (2004) did not find a significant association between FA and other measures of individual fitness and allometry in the bat genitalia. All previous studies analyzing humeral asymmetry in bats were based on adult specimens with fully ossified epiphyses, whereas our study could not analyze cartilaginous osseous tissue.

Studies have also linked the presence and magnitude of FA to anthropogenic perturbations and habitat degradation, analyzing four Neotropical phyllostomid species (i.e., *Artibeus lituratus*, *Artibeus planirostris*, *C. perspicillata*, and *Sturnira lilium*) (de Figueiredo et al., 2015; Castillo-Figueroa, 2018). Neither study explicitly tested the presence of the three kinds of asymmetry, casting doubt on their interpretation that FA is an accurate index of resilience and adaptation of species to perturbations. Robaina et al. (2017) may have also insufficiently assessed the presence and magnitude of all types of asymmetry before drawing conclusions on the validity of FA to reflect functional importance. Our results suggest that special attention to the statistical framework used to describe the biological and theoretical interpretation of asymmetry in bats is warranted.

Asymmetry across multiple traits has shown decoupled patterns, reflecting functional differences and indicating independent developmental mechanisms controlling phenotypic symmetry across different structures of the body of bats (Gummer and Brigham, 1995; Robaina et al., 2017), turtles (Rivera and Neely, 2020), and birds (Aparicio and Bonal, 2002). Our results of decoupled patterns of longitudinal and cross-sectional humeral FA point toward independent trajectories of bone elongation (endochondral ossification) and thickening (intramembranous ossification) while also suggesting that it could be applicable within single structures. Longitudinal and cross-sectional growth are hypothesized to be decoupled ontogenetic processes (Enlow, 1963; Montoya-Sanhueza et al., 2019) and to respond to different selective pressures, with cross-sectional bone deposition potentially associated with biomechanical resistance against torsional and bending stresses (Blackburn, 2011; Perchalski et al., 2018) and bone elongation correlating with maintenance of body proportions within a functional unit (Ueti et al., 2015).

Peaks of longitudinal (stages 2 and 6) and cross-sectional FA (stage 5) at different developmental stages could indicate variations in the timing of ossification onset and/or growth rates across species, following the morphogenetic drift model (Hallgrímsson, 1998). Despite bats showing a general mammalian developmental pattern (López-Aguirre et al., 2019a), heterochronies and diverging allometric trajectories for the ossification of the humeri have been found across bat species (López-Aguirre et al., 2019b). The reported slower ossification of the humerus in yinpterochiropteran bats could indicate that different growth rates at a given developmental stage could result in increased magnitudes of FA (López-Aguirre et al., 2019b). However, our study does not allow for the accurate comparison of growth rates across species, reflecting the challenge of amassing embryonic material for non-model species, as not all species have complete developmental series. Future studies should focus on further exploring the relationship between interspecific variation in growth rates and magnitudes of FA. Despite both longitudinal and cross-sectional FA showing homogenous magnitudes across development, only cross-sectional FA showed statistically significant differences across developmental stages. Increasing postcranial morphological disparity and integration across prenatal development in bats has been reported in ossification sequences and metric growth (López-Aguirre et al., 2019a).

We hypothesize that our results (significant differences in cross-sectional FA across stages) indicate a greater selective pressure to canalize cross-sectional asymmetry as a response to functional demands associated with a newborn bat’s ecology. In particular, there may be a greater requirement for symmetrical cross-sectional growth to facilitate early roosting behaviors. In many bat species, newborns attach to the mothers using their feet and thumb (Koyabu and Son, 2014) rather than to immediately fly, with bone elongation continuing during this period. Multiple studies have documented postnatal development of flight in bats, describing the altricial development of the forelimb in bats followed by accelerated bone elongation (Hughes and Rayner, 1993; Kunz and Robson, 1995; Kunz et al., 2009; Lin et al., 2010; Eghbali et al., 2017; Eghbali and Sharifi, 2018). First flights in most species that have been studied occur consistently in synchrony with weaning, usually a couple of weeks after birth once adult body dimensions are reached (Hughes and Rayner, 1993; Kunz and Robson, 1995; Kunz et al., 2009; Lin et al., 2010; Eghbali et al., 2017; Eghbali and Sharifi, 2018). We hypothesize that because self-powered flight is not achieved immediately after birth, bone elongation asymmetry and compensatory growth to optimize wing proportions would be less constrained prenatally (Ueti et al., 2015). Further studies quantifying the developmental trajectories of asymmetry should focus on describing the complementary developmental process (pre- and postnatal). Additionally, testing this hypothesis in mammal species with newborns that also cling to the mothers (e.g., macaques and colugos) would further elucidate whether the patterns found in this study are common across Mammalia.

We found significant support for the presence of FA and DA during the prenatal development of the humerus in bats. We also show that magnitudes of FA remain stable across prenatal development, and we hypothesize this to be evidence of developmental control of FA. Moreover, we find evidence for decoupled patterns of longitudinal and cross-sectional asymmetry throughout prenatal humeral development. We suggest that decoupled morphogenetic processes (i.e., bone thickening *via* intramembranous ossification and bone elongation *via* endochondral ossification) and functional differences between bone elongation and cross-sectional bone deposition may be associated with the newborn’s ecology (i.e., pup roosting behavior and the later acquisition of flight). To our knowledge, this study is the first to analyze asymmetry patterns in the development of bats, providing new information about phenotypic asymmetry and DI in non-model taxa. We highlight the importance of assessing the correlation between FA and DI beyond patterns of total asymmetry FA and DI.

## Data Availability Statement

The original contributions presented in the study are included in the article/Supplementary Material, further inquiries can be directed to the corresponding author/s.

## Ethics Statement

The animal study was reviewed and approved by the Institute of Ecology and Biological Resources, Vietnam Academy of Sciences ethics permit No. 322/STTNSV.

## Author Contributions

CL-A, SH, and LW designed the study. CL-A gathered and analyzed the data. CL-A, SH, LW, and DK wrote the manuscript. DK and VTT sampled the specimens. DK prepared and scanned the specimens. CL-A processed the scans. All authors read and approved the final manuscript.

## Conflict of Interest

The authors declare that the research was conducted in the absence of any commercial or financial relationships that could be construed as a potential conflict of interest.

## References

[B1] Adams D. C. Otárola-Castillo E. Paradis E. (2013). Geomorph: an R package for the collection and analysis of geometric morphometric shape data. *Methods Ecol. Evol.* 4 393–399. 10.1111/2041-210x.12035

[B2] Adams R. A. (1992). Comparative skeletogenesis of the forearm of the little brown bat (*Myotis-Lucifugus*) and the Norway Rat (*Rattus-Norvegicus*). *J. Morphol.* 214 251–260. 10.1002/jmor.1052140302 1474596

[B3] Adams R. A. (2008). Morphogenesis in bat wings: linking development, evolution and ecology. *Cells Tissues Organs* 187 13–23. 10.1159/000109960 18163246

[B4] Adams R. A. Shaw J. B. (2013). “Time’s arrow in the evolutionary development of bat flight,” in *Bat Evolution, Ecology, and Conservation*, eds Adams R. Pedersen S. (New York, NY: Springer), 21–46. 10.1007/978-1-4614-7397-8_2

[B5] Allard P. Tabin C. J. (2009). Achieving bilateral symmetry during vertebrate limb development. *Semin. Cell Dev. Biol.* 20 479–484. 10.1016/j.semcdb.2008.10.011 19027866

[B6] Aparicio J. (1998). Patterns of fluctuating asymmetry in developing primary feathers: a test of the compensational growth hypothesis. *Proc. R. Soc. Lond. Ser. B Biol. Sci.* 265 2353–2357. 10.1098/rspb.1998.0583

[B7] Aparicio J. M. Bonal R. (2002). Why do some traits show higher fluctuating asymmetry than others? A test of hypotheses with tail feathers of birds. *Heredity* 89 139–144. 10.1038/sj.hdy.6800118 12136417

[B8] Atchley W. R. Hall B. K. (1991). A model for development and evolution of complex morphological structures. *Biol. Rev.* 66 101–157. 10.1111/j.1469-185X.1991.tb01138.x 1863686

[B9] Blackburn A. (2011). Bilateral asymmetry of the humerus during growth and development. *Am. J. Phys. Anthropol.* 145 639–646. 10.1002/ajpa.21555 21702005

[B10] Broek C. M. A. T. Bots J. Bugiani M. Galis F. Dongen S. V. (2017). Developmental origins of limb developmental instability in human fetuses: many abnormalities make the difference. *Symmetry* 9:51. 10.3390/sym9040051

[B11] Brown E. E. Cashmore D. D. Simmons N. B. Butler R. J. (2019). Quantifying the completeness of the bat fossil record. *Palaeontology* 62 757–776. 10.1111/pala.12426

[B12] Camacho J. Heyde A. Bhullar B-A. S. Haelewaters D. Simmons N. B. Abzhanov A. (2019). Peramorphosis, an evolutionary developmental mechanism in neotropical bat skull diversity. *Dev. Dyn.* 248, 1129–1143. 10.1002/dvdy.90 31348570

[B13] Castillo-Figueroa D. (2018). Fluctuating asymmetry of three bat species in extensive livestock systems from Córdoba department, Colombia. *Rev. Colomb. Ciencia Animal Recia* 10 143–153. 10.24188/recia.v10.n2.2018.623

[B14] Cooper L. Cretekos C. J. Sears K. E. (2012). The evolution and development of mammalian flight. *Wiley Interdiscip. Rev. Dev. Biol.* 1 773–779. 10.1002/wdev.50 23799572

[B15] Cretekos C. J. Wang Y. Green E. D. Martin J. F. Rasweiler J. J. T. Behringer R. R. (2008). Regulatory divergence modifies limb length between mammals. *Genes Dev.* 22 141–151. 10.1101/gad.1620408 18198333 PMC2192750

[B16] Cretekos C. J. Weatherbee S. D. Chen C. H. Badwaik N. K. Niswander L. Behringer R. R. (2005). Embryonic staging system for the short-tailed fruit bat, *Carollia perspicillata*, a model organism for the mammalian order Chiroptera, based upon timed pregnancies in captive-bred animals. *Dev. Dyn.* 233 721–738. 10.1002/dvdy.20400 15861401

[B17] de Figueiredo D. Gonçalves Miller B. Barbosa Leal E. S. Montes M. A. (2015). Fluctuating asymmetry in populations of bats: species adapted to urban environments are not hampered by habitat degradation. *Chiropt. Neotrop.* 21 1305–1311.

[B18] Dongen S. V. (2006). Fluctuating asymmetry and developmental instability in evolutionary biology: past, present and future. *J. Evol. Biol.* 19 1727–1743. 10.1111/j.1420-9101.2006.01175.x 17040371

[B19] Dumont E. R. (2010). Bone density and the lightweight skeletons of birds. *Proc. R. Soc. B Biol. Sci.* 277 2193–2198. 10.1098/rspb.2010.0117 20236981 PMC2880151

[B20] Eghbali H. Shahabi S. Najafi N. Mehdizadeh R. Yousefi S. Sharifi M. (2017). Postnatal growth, wing development and age estimations in the Mediterranean horseshoe bat *Rhinolophus* euryale (Chiroptera: Rhinolophidae) in Kerend cave, western Iran. *Mammalia* 82 276–287. 10.1515/mammalia-2017-0006

[B21] Eghbali H. Sharifi M. (2018). Postnatal growth, age estimation, and wing development in Geoffroy’s Bat *Myotis emarginatus* (Chiroptera: Vespertilionidae). *Mammal Study* 43 153–165. 10.3106/ms2017-0077

[B22] Enlow D. H. (1963). *Principles of Bone Remodeling: An Account of Post-natal Growth and Remodeling Processes in Long Bones and the Mandible.* Springfield, IL: Thomas.

[B23] Farnum C. E. Tinsley M. Hermanson J. W. (2008). Forelimb versus Hindlimb Skeletal development in the big brown bat, *Eptesicus fuscus:* functional divergence is reflected in chondrocytic performance in autopodial growth plates. *Cells Tissues Organs* 187 35–47. 10.1159/000109962 18160801

[B24] Gummer D. L. Brigham R. M. (1995). Does fluctuating asymmetry reflect the importance of traits in little brown bats (*Myotis lucifugus*)? *Can. J. Zool.* 73 990–992. 10.1139/z95-116

[B25] Hallgrímsson B. (1998). Fluctuating asymmetry in the mammalian skeleton: evolutionary and developmental implications. *Evol. Biol.* 30 187–251. 10.1007/978-1-4899-1751-5_6

[B26] Hallgrímsson B. (1999). Ontogenetic patterning of skeletal fluctuating asymmetry in rhesus macaques and humans: evolutionary and developmental implications. *Int. J. Primatol.* 20 121–151. 10.1023/A:1020540418554

[B27] Hallgrímsson B. Miyake T. Wilmore K. Hall B. K. (2003). Embryological origins of developmental stability: size, shape and fluctuating asymmetry in prenatal random bred mice. *J. Exp. Zool. Part B Mol. Dev. Evol.* 296 40–57. 10.1002/jez.b.15 12658710

[B28] Hughes P. Rayner J. M. V. (1993). The flight of pipistrelle bats *Pipistrellus pipistrellus* during pregnancy and lactation. *J. Zool.* 230 541–555. 10.1111/j.1469-7998.1993.tb02705.x

[B29] Juste J. López-González C. Strauss R. E. (2001a). Analysis of asymmetries in the African fruit bats Eidolon helvum and *Rousettus egyptiacus* (Mammalia: Megachiroptera) from the islands of the Gulf of Guinea. I. Variance and size components of bilateral variation. *J. Evol. Biol.* 14 663–671. 10.1046/j.1420-9101.2001.00298.x

[B30] Juste J. López-González C. Strauss R. E. (2001b). Analysis of asymmetries in the African fruit bats Eidolon helvum and *Rousettus egyptiacus* (Mammalia: Megachiroptera) from the islands of the Gulf of Guinea. II. Integration and levels of multivariate fluctuating asymmetry across a geographical range. *J. Evol. Biol.* 14 672–680. 10.1046/j.1420-9101.2001.00299.x

[B31] Kellner J. R. Alford R. A. (2003). The ontogeny of fluctuating asymmetry. *Am. Natural.* 161 931–947. 10.1086/375177 12858277

[B32] Klingenberg C. P. (2015). Analyzing fluctuating asymmetry with geometric morphometrics: concepts, methods, and applications. *Symmetry* 7 843–934. 10.3390/sym7020843

[B33] Klingenberg C. P. McIntyre G. S. (1998). Geometric morphometrics of developmental instability: analyzing patterns of fluctuating asymmetry with procrustes methods. *Evolution* 52 1363–1375. 10.1111/j.1558-5646.1998.tb02018.x 28565401

[B34] Klingenberg C. P. Nijhout H. F. (1999). Genetics of fluctuating asymmetry: a developmental model of developmental instability. *Evolution* 53 358–375. 10.1111/j.1558-5646.1999.tb03772.x 28565420

[B35] Koyabu D. Son N. (2014). Patterns of postcranial ossification and sequence heterochrony in bats: life histories and developmental trade-offs. *J. Exp. Zool. Part B Mol. Dev. Evol.* 322 607–618. 10.1002/jez.b.22581 24863050

[B36] Kronenberg H. M. (2003). Developmental regulation of the growth plate. *Nature* 423 332–336. 10.1038/nature01657 12748651

[B37] Kunz T. H. Adams R. A. Wood W. R. (2009). “Methods for assessing size at birth and postnatal growth and development in bats,” in *Ecological and behavioral methods for the study of bats*, eds Kunz T. H. Parsons S. (Baltimore, MD: The Johns Hopkins University Press), 273–314.

[B38] Kunz T. H. Robson S. K. (1995). Postnatal growth and development in the mexican free-tailed bat (*Tadarida brasiliensis* mexicana): birth size, growth rates, and age estimation. *J. Mammal.* 76 769–783. 10.2307/1382746

[B39] Leamy L. J. Klingenberg C. P. (2005). The genetics and evolution of fluctuating asymmetry. *Annu. Rev. Ecol. Evol. Syst.* 36 1–21. 10.1146/annurev.ecolsys.36.102003.152640

[B40] Lin A. Jin L. Liu Y. Sun K. Feng J. X. (2010). Postnatal growth and age estimation in Horsfield’s Leaf-Nosed Bat *Hipposideros larvatus*. *Zool. Stud.* 49 789–796.

[B41] López-Aguirre C. Hand S. J. Koyabu D. Son N. T. Wilson L. A. B. (2019a). Postcranial heterochrony, modularity, integration and disparity in the prenatal ossification in bats (Chiroptera). *BMC Evol. Biol.* 19:75. 10.1186/s12862-019-1396-1 30866800 PMC6417144

[B42] López-Aguirre C. Hand S. J. Koyabu D. Son N. T. Wilson L. A. B. (2019b). Prenatal allometric trajectories and the developmental basis of postcranial phenotypic diversity in bats (Chiroptera). *J. Exp. Zool. Part B Mol. Dev. Evol.* 332 36–49. 10.1002/jez.b.22846 30793502

[B43] López-Aguirre C. Pérez-Torres J. (2015). Asimetría cráneo-mandibular de Artibeus lituratus (Chiroptera, Phyllostomidae) en Colombia. *Univ. Scientiarum* 20 141–152. 10.11144/javeriana.sc20-1.acal

[B44] López-Aguirre C. Wilson L. A. Koyabu D. Tan Tu V. Hand S. J. (2019). Decoupled morphological and biomechanical evolution and diversification of the wing in bats. *EcoEvoRxiv* 31 1–31. 10.32942/osf.io/k3y5f

[B45] Lüpold S. McElligott A. G. Hosken D. J. (2004). Bat genitalia: allometry, variation and good genes. *Biol. J. Linn. Soc.* 83 497–507. 10.1111/j.1095-8312.2004.00407.x

[B46] Macintosh A. A. Davies T. G. Ryan T. M. Shaw C. N. Stock J. T. (2013). Periosteal versus true cross-sectional geometry: a comparison along humeral, femoral, and tibial diaphyses. *Am. J. Phys. Anthropol.* 150 442–452. 10.1002/ajpa.22218 23359138

[B47] Monteiro L. R. Mellado B. Nogueira M. R. de Morais-Jr M. M. (2019). Individual asymmetry as a predictor of fitness in the bat *Carollia perspicillata*. *J. Evol. Biol.* 32 1207–1229. 10.1111/jeb.13522 31420901

[B48] Montoya-Sanhueza G. Wilson L. A. B. Chinsamy A. (2019). Postnatal development of the largest subterranean mammal (*Bathyergus suillus*): morphology, osteogenesis, and modularity of the appendicular skeleton. *Dev. Dyn.* 248 1101–1128. 10.1002/dvdy.81 31265186

[B49] Palmer A. R. (1994). “Fluctuating asymmetry analyses: a primer,” in *Developmental Instability: Its Origins and Evolutionary Implications: Proceedings of the International Conference on Developmental Instability: Its Origins and Evolutionary Implications, Tempe, Arizona*, ed. Markow T. A. (Dordrecht: Springer), 335–364. 10.1007/978-94-011-0830-0_26

[B50] Panyutina A. Korzun L. P. Kuznetsov A. N. (2015). *Flight of Mammals: From Terrestrial Limbs to Wings.* New York, NY: Springer.

[B51] Papadimitriou H. M. Swartz S. M. Kunz T. H. (1996). Ontogenetic and anatomic variation in mineralization of the wing skeleton of the Mexican free-tailed bat, *Tadarida brasiliensis*. *J. Zool.* 240 411–426. 10.1111/j.1469-7998.1996.tb05295.x

[B52] Perchalski B. Placke A. Sukhdeo S. M. Shaw C. N. Gosman J. H. Raichlen D. A. (2018). Asymmetry in the cortical and trabecular bone of the human humerus during development. *Anat. Rec. (Hoboken)* 301 1012–1025. 10.1002/ar.23705 29055969

[B53] Rivera G. Neely C. M. D. (2020). Patterns of fluctuating asymmetry in the limbs of freshwater turtles: are more functionally important limbs more symmetrical? *Evolution* 74 660–670. 10.1111/evo.13933 31989579

[B54] Robaina Z. Denis D. Sánchez-Lozada M. (2017). ¿Son regulados los niveles de asimetría fluctuante según la función de las estructuras morfológicas? Estudio de caso en Phyllonycteris poeyi (chiroptera: phyllostomidae)/Are levels of fluctuating asymmetry regulated according to the function of. *Poeyana* 505 15–21.

[B55] Sears K. E. Behringer R. R. Rasweiler J. J. Niswander L. A. (2006). Development of bat flight: morphologic and molecular evolution of bat wing digits. *Proc. Natl. Acad. Sci. U.S. A.* 103 6581–6586. 10.1073/pnas.0509716103 16618938 PMC1458926

[B56] Spoor C. F. Zonneveld F. W. Macho G. A. (1993). Linear measurements of cortical bone and dental enamel by computed-tomography - applications and problems. *Am. J. Phys. Anthropol.* 91 469–484. 10.1002/ajpa.1330910405 8372936

[B57] Swaddle J. P. Witter M. S. (1997). On the ontogeny of developmental stability in a stabilized trait. *Proc. R. Soc. Lond. B Biol. Sci.* 264 329–334.

[B58] Swartz S. M. (1997). Allometric patterning in the limb skeleton of bats: implications for the mechanics and energetics of powered flight. *J. Morphol.* 234 277–294. 29852636 10.1002/(SICI)1097-4687(199712)234:3<277::AID-JMOR6>3.0.CO;2-6

[B59] Swartz S. M. Bennett M. B. Carrier D. R. (1992). Wing bone stresses in free flying bats and the evolution of skeletal design for flight. *Nature* 359 726–729. 1436035 10.1038/359726a0

[B60] Tocts A. M. S. Johnson D. W. Carter A. J. R. (2016). Strong nonlinear selection against fluctuating asymmetry in wild populations of a marine fish. *Evolution* 70 2899–2908. 10.1111/evo.13092 27757960

[B61] Ueti A. Pompeu P. S. Ferreira R. L. (2015). Asymmetry compensation in a small vampire bat population in a cave: a case study in Brazil. *Subterranean Biol.* 15 57–67. 10.3897/subtbiol.15.4807

[B62] Voigt C. C. Heckel G. Mayer F. (2005). Sexual selection favours small and symmetric males in the polygynous greater sac-winged bat *Saccopteryx bilineata* (Emballonuridae, Chiroptera). *Behav. Ecol. Sociobiol.* 57 457–464. 10.1007/s00265-004-0874-6

[B63] Webber T. Patel S. P. Pensak M. Fajolu O. Rozental T. D. Wolf J. M. (2015). Correlation between distal radial cortical thickness and bone mineral density. *J. Hand Surg.* 40 493–499. 10.1016/j.jhsa.2014.12.015 25708436

[B64] Wilson L. Humphrey L. T. (2015). A Virtual geometric morphometric approach to the quantification of long bone bilateral asymmetry and cross-sectional shape. *Am. J. Phys. Anthropol.* 158 541–556. 10.1002/ajpa.22809 26208153

[B65] Wolpert L. (2010). Arms and the man: the problem of symmetric growth. *PLoS Biol.* 8:e1000477. 10.1371/journal.pbio.1000477 20838659 PMC2935459

