# Supplementary figures and images for: Prenatal Developmental Trajectories of Fluctuating Asymmetry in Bat Humeri
Source: Front Cell Dev Biol. 2021 May 26;9:639522. doi: 10.3389/fcell.2021.639522 (PMC8187808; doi:10.3389/fcell.2021.639522)

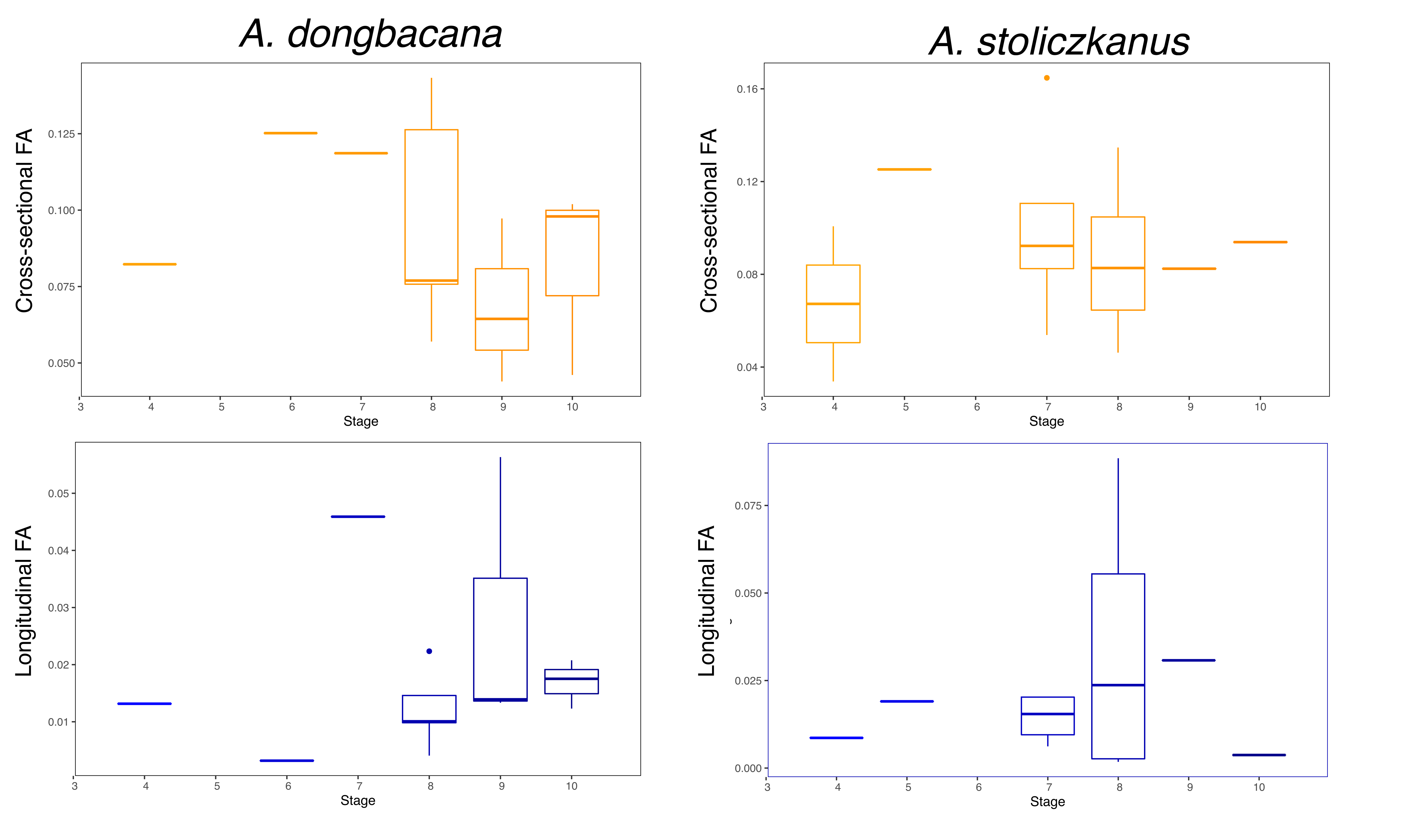

Supplement: Supplementary file 2 [file Image_1.PNG]

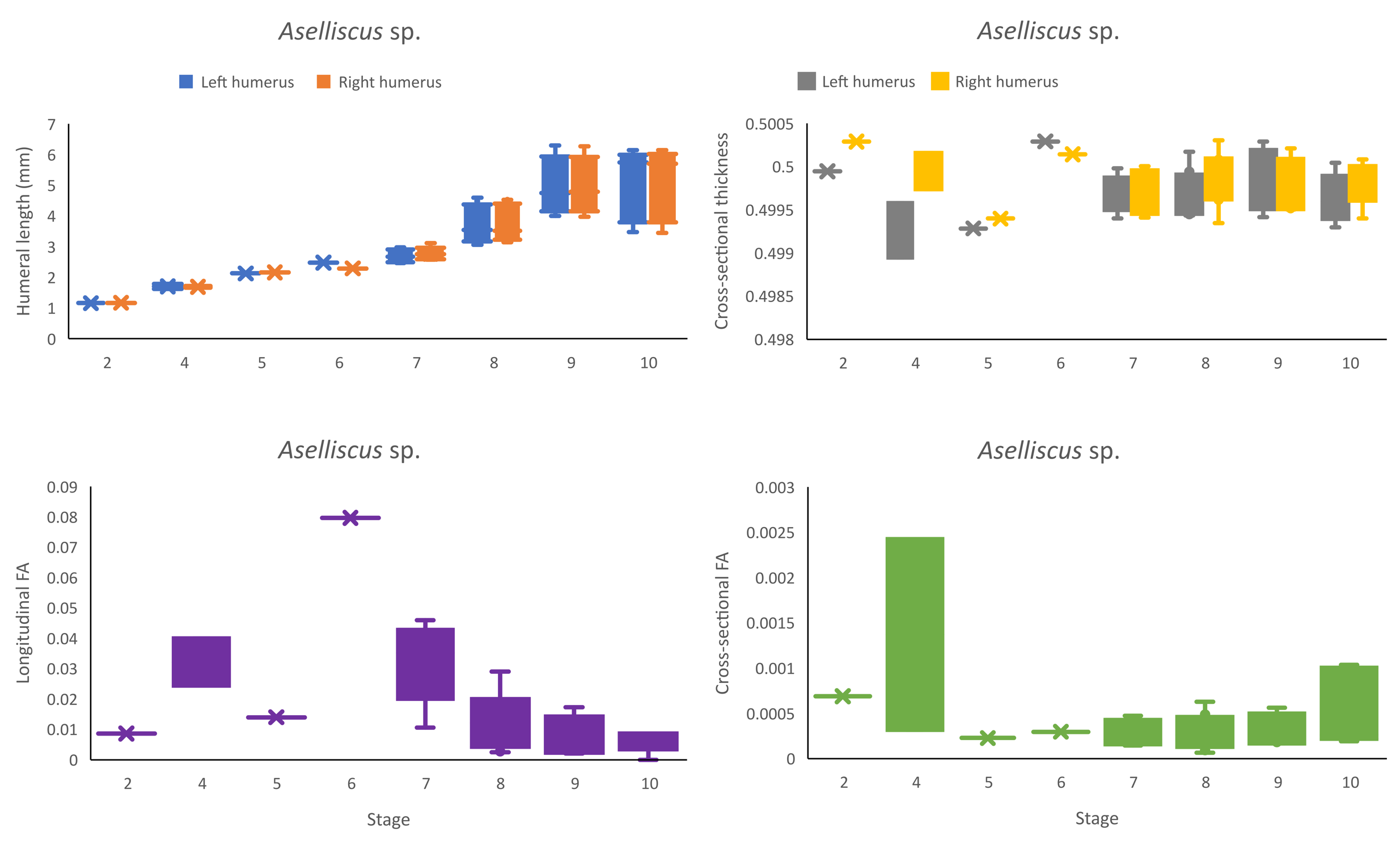

Supplement: Supplementary file 3 [file Image_2.PNG]
